# Remarks on Medical Reform, in a Letter Addressed to the Right Hon. Sir James Graham, Bart. &c.

**Published:** 1843-04

**Authors:** 


### ART. XXI.

1. *Remarks on Medical Reform, in a Letter addressed to the Right Hon. Sir James Graham, Bart., one of Her Majesty's Principal Secretaries of State, &c.* By Sir JAMES CLARK, Bart., M.D. F.R.S., Physician to the Queen and the Prince Albert.—*London*, June, 1842. 8vo, pp. 30.

2. *Remarks on Medical Reform, in a Second Letter addressed to Sir James Graham, &c.* By Sir JAMES CLARK, Bart., M.D. F.R.S. &c.—*London*, March, 1843. 8vo, pp. 42.

SIR JAMES CLARK has always been the strenuous and consistent advocate of Medical Reform; and, in all his writings on this subject, he has dwelt especially on the importance of improving the education of medical men, as the fundamental principle of all rational change. He is indeed a strong advocate for corporate reform, also, and the remodelling the existing institutions, so as to make them accessible to the great body of medical men, and not to a class only; but these measures he regards as secondary to that individual vital reform, which consists in improving and enlarging the minds of those who are to constitute the future members of the profession. So early as the years 1822 and 1823, we find Sir James advocating the cause of medical education and, at the same time, defending the mode of teaching in his *alma mater*, and English medical literature generally, against the attacks of Tommasini, in his Letters published at Rome, and addressed to that celebrated professor in his own language.* And at a subsequent period we find him endeavouring to improve the very school he had defended, by pointing out the advantages it would derive from adopting some of the foreign modes of instruction, the value of which his travels and long sojourn on the continent had enabled him to appreciate.† It will not be supposed, therefore, that the measures of reform, proposed or advocated by our author, are either visionary or lightly considered. They are well worthy the attention of the profession; and we hope they will receive mature consideration from the statesman to whom they are especially addressed.

In the first of the pamphlets now before us, that published last year, Sir James Clark pressed upon the attention of the Home Secretary, the especial claims the general practitioner had to be considered in any legislative enactment for improving the profession; he showed "that the establishment of a good and uniform system of education, applicable to all candidates for licences to practise, was the primary object to be kept in view in any scheme of Medical Reform." He recommended "the union of the Colleges of Physicians and Surgeons, in order to embrace in one great corporate institution all the members of the profession." He pointed out how "this union might be effected without disturbing the distinctions and grades which are recognized in the profession, whilst it could not fail

* Lettera di Giacomo Clark, M.D. al. ch. sig. Prof. Tommasini intorno alle sue osservazioni sulla scuola medico-clinica di Edinburgo—Roma, 1822.

Lettera del dottore Giacomo Clark al. Prof. Giacomo Tommasini intorno alla Letteratura medico Inglese.—Roma, 1823.

† Observations on the System of Teaching Clinical Medicine in the University of Edinburgh, with Suggestions for its Improvement.—London, 1827. 8vo.

to raise the character of medical practitioners generally, promote harmony among them, and advance the cause of medical science." He expressed his "belief that the continuance of the Apothecaries' Company would be injurious to the profession, and that the practice of pharmacy ought to be separated from that of medicine;" and he predicted "that little pernent advantage could result to the present Colleges from any change in their constitution, which had not for its direct aim and object the good of the whole profession."

He says that the principal object he has in view in addressing the Home Secretary on the present occasion, "is to urge the necessity of so framing the legislative enactment which he is about to introduce, as thereby to secure to all who are permitted to engage in the practice of medicine a GOOD EDUCATION. This (the author truly adds,) is unquestionably the most important part of what is implied by Medical Reform, and that calling most loudly for the interference of the legislature. Until it is obtained, any scheme of reforming or modifying the existing medical corporations will be productive of little benefit to the profession, and less to the public."

To enable Sir James Graham fully to understand the subject on which he is about to legislate, Sir James Clark takes a review of the history of the profession during the last fifty years, and then delineates "the actual state and relative position of medical practitioners in this country." In this survey he shows how the apothecary "from being the humble individual whose duty it was implicitly to follow the directions of the physician, and compound the drugs which he prescribed, has gradually risen to be the ordinary medical attendant of the great bulk of the population; and, for the most part, is now only required to summon the physician to his aid in cases of difficulty or danger;" and he justly asks, "Ought any man to be intrusted with such duties who has not brought a well-instructed and disciplined mind to the study of a profession involving such vital interests? And is it not the duty of the legislature to take care that no man shall be licensed to undertake these duties without having adduced proofs of being qualified to perform tbem?"

Notwithstanding the great improvement in the education and position of the general practitioner, Sir James Clark considers his education to be still very defective, "more especially in its preliminary or general part;" and he says that it is "to bring his education up to the standard of his responsibilities," that legislative interference is now especially required. He considers fully all the objections that have been advanced against improving the preliminary education of the general practitioner, and shows, in our opinion, the perfect futility of the whole. To enable students to compass this superior education, he advocates, of course, the abolishment of the degrading system of apprenticeships, and agrees with Sir Benjamin Brodie in the propriety of raising the age at which the licence to practise is obtainable. Sir James Clark considers it essential, in opposition to the opinion of many, that "the general or preliminary instruction should be completed before the professional education commences;" and here we think he is obviously right; for the very object of the preliminary education is the fixing of a basis on which professional knowledge can be erected. The student, of course, may if he pleases, continue—and it is to be hoped that he will continue—to prosecute his more general along with his strictly professional studies; but the time requisite for the latter must not be encroached on by the former.

Our space does not permit us to enter upon the consideration of various other points treated of in Sir James Clark's Letter; but we feel assured that if the measure about to be introduced by Sir James Graham, does not embrace the main features advocated in it, the profession will have little occasion to be satisfied with the new arrangement. But we confess that for our parts, we have little fear on the subject. It is impossible that the general practitioner can be overlooked in any measure intended to improve the medical profession ; to do so were indeed to play Hamlet with the part of Hamlet left out. We think it equally unlikely that the Colleges of Physicians and Surgeons can be put on a new footing, without the abolition of their present exclusiveness and irresponsibility; and if there is to be constituted, as we are informed, a Central Board of Control for the regulation of medical education, and the establishment of tribunals for testing the proficiency of candidates, we have too much faith in the power of publicity and of the press, to doubt, that the regulations thence emanating for the government of the profession in its new condition, will be such as are in unison with the liberal spirit of the times, and can be cheerfully submitted to by the enlightened cultivators and professors of a science and art second to none in dignity and usefulness.

---

